# New Morphological Features for Grading Pancreatic Ductal Adenocarcinomas

**DOI:** 10.1155/2013/175271

**Published:** 2013-07-25

**Authors:** Jae-Won Song, Ju-Hong Lee

**Affiliations:** Department of Computer & Information Engineering, Inha University, 253 Yonghyun-dong, Nam-gu, Incheon 402-751, Republic of Korea

## Abstract

Pathological diagnosis is influenced by subjective factors such as the individual experience and knowledge of doctors. Therefore, it may be interpreted in different ways for the same symptoms. The appearance of digital pathology has created good foundation for objective diagnoses based on quantitative feature analysis. Recently, numerous studies are being done to develop automated diagnosis based on the digital pathology. But there are as of yet no general automated methods for pathological diagnosis due to its specific nature. Therefore, specific methods according to a type of disease and a lesion could be designed. This study proposes quantitative features that are designed to diagnose pancreatic ductal adenocarcinomas. In the diagnosis of pancreatic ductal adenocarcinomas, the region of interest is a duct that consists of lumen and epithelium. Therefore, we first segment the lumen and epithelial nuclei from a tissue image. Then, we extract the specific features to diagnose the pancreatic ductal adenocarcinoma from the segmented objects. The experiment evaluated the classification performance of the SVM learned by the proposed features. The results showed an accuracy of 94.38% in the experiment distinguishing between pancreatic ductal adenocarcinomas and normal tissue and a classification accuracy of 77.03% distinguishing between the stages of pancreatic ductal adenocarcinomas.

## 1. Introduction

Pathological diagnosis is currently performed subjectively via the knowledge and experience of doctors after inspection of tissue slides through light microscopes. This subjective diagnosis has some problems. First, tumor screening at high magnification by light microscope requires a lot of time and effort [1, 2]. Also, the individual competence of doctors has a decisive effect on the final diagnosis. This means that the pathological diagnosis of the same tumor by two different doctors may vary, because it is not based on objective quantitative feature analysis [3–5]. 

The advent of digital pathology has led to a new type of pathological diagnosis. Digital pathology changes glass slides into digital images. It provides a convenient screening environment that also makes it possible to objectively diagnose tumors through quantitative feature analysis using a computer [6–8]. Nevertheless, a pathological diagnosis needs much time and workload because the diagnosis is manually processed. Therefore, there are still problems related to the subjective diagnosis of tumors. To overcome these problems, studies on computer-aided diagnosis (CAD) techniques based on digital pathology are growing. Many studies of CAD-based digital pathology have covered not only detection of tumors but also grading the stages of tumors. In pathology, tumor stage grading is useful to identify the extent of the disease and determine the appropriate treatment for a patient [9]. In fact, it is known that pathologists receive intensive trainings on grading stages of tumors in order to prescribe the correct type of treatment [7]. Currently, many studies of pathological diagnosis with CAD techniques are being carried out on breast and prostate tumors.

First, most CAD studies related to prostate cancer are based on the Gleason grading system [10]. Tabesh et al. [11] proposed an automated system based on machine learning to diagnose prostate cancer and grade the stages (low and high) of cancer with the Gleason grading system. They extracted color, texture, and morphometric features at global and object levels of a given tissue image. Classifying algorithms such as Gaussian, *k*-nearest neighbor (*k*-NN), and Support Vector Machine (SVM) learned the features of cancer diagnosis and Gleason grading. Naik et al. [12] proposed a diagnostic system for distinguishing between intermediate Gleason grades. They identified the candidate gland region using a Bayesian classifier with low-level information and eliminated false positive regions identified as glands using empirical domain information. After that, the morphologic features were extracted from the identified glands, and Gleason grades 3, 4, and benign were classified through the SVM that learned the features. Huang and Lee [13] classified prostate cancer images into 4 grades based on a Gleason grading system. They used Bayesian, *k*-NN, and SVM classifiers for classifying stages of the cancer. And, to teach the classifiers Gleason grading, features are proposed by using differential box-counting and entropy-based dimension estimation techniques. In addition to that, there are many studies for the grading and diagnosis of prostate tumors [14–17].

There are also CAD studies related to breast tumors. Anderson et al. [18] worked on a problem for distinguishing ductal hyperplasia (DH), which is benign, and ductal carcinoma in situ (DCIS), which is malignant. In this study, they automatically segmented breast ducts using knowledge-guided machine vision and proposed measuring duct cribriformity and architectural complexity to quantitatively analyze the duct patterns in proliferative lesions to distinguish between DH and DCIS. Bilgin et al. [19] proposed a method to diagnose breast cancer using graph theory techniques. They segmented given tissue images using a *k*-means algorithm and generated different cell graphs using the positional coordinates of cells for each segmented image. An SVM model that can classify given tissue images into benign, invasive, and non-invasive (ductal carcinoma *in situ*) was learned by quantitative metrics that are computed from the generated cell graphs. Basavanhally et al. [20] proposed a grading system that identifies and grades the extent of lymphocytic infiltration (LI), a known viable prognostic indicator. First, they detected lymphocytes using region growing and Markov random field algorithms. Then, the architectural features were extracted from the detected lymphocytes, and the extent of LI was classified into low, medium, and high grades by an SVM classifier learned by the features. In addition, there are a lot of studies for the diagnosis and grading of breast tumors [21–24].

Many pathological CAD studies make an effort to analyze the pathological characteristics of and design the methods for quantitatively measuring each disease, because there are many methods for pathological diagnosis according to the type of disease and lesion. Currently, in addition to the breast and prostate tumors mentioned above, some studies on colonic [25], bladder [26], neuroblastoma [27–29], and follicular lymphoma [30, 31] tumors have been performed. Pathological CAD studies are still in the early stages and focus on a few tumors. There are many methods for diagnosing different types of tumors. Therefore, more studies of pathological CADs must be performed. 

The aim of this study is to detect pancreatic ductal adenocarcinoma (PDAC) and classify them by stages. To achieve this, we propose new morphological features for diagnosing and grading PDAC. The region that is inspected to diagnose PDAC is a duct that consists of lumen and epithelium. Therefore, this paper segments the given image into lumen, epithelial nuclei, and nonepithelial nuclei and extracts the morphological features for diagnosing PDAC from the segmented objects. After that, the diagnosis and grading stages of PDAC are performed using the SVM model learned by the extracted features. This paper has several sections.  Section 2 describes the pathological characteristics and the morphological features needed in diagnosing PDAC.  Section 3 shows the configuration of systems used to diagnose PDAC and the segmentation methods of objects.  Section 4 discusses the proposed new morphological features for quantitatively measuring the pathological characteristics of PDAC described in Section 2. In Section 5, the performances between SVM classifiers learnt by the proposed and existing classical morphological features are compared with each other to show the suitability of the proposed features to detect and grade the stages of PDAC.  Section 6 evaluates and statistically analyzes the results. Finally, Section 7 presents a conclusion of this study.

## 2. Pathological Characteristics of PDAC

Pancreatic cancer is the second most common gastrointestinal neoplasm that causes death, after colon cancer [33]. And of all pancreatic neoplasms, PDAC accounts for 85–95%. Approximately 80% of all PDAC patients are between 60 and 80 years of age, and cases in people below the age of 40 are rare. The incidence of PDAC is about 50% higher in men than in women. By race, those of African ancestry have the highest rate of PDAC [34]. The best way of treating PDAC is known as curative resection. However, because of the rare possibility of diagnosing PDAC in the early stage, only 5–22% of PDAC patients can take the curative resection at the time the cancer is discovered [35]. Therefore, an accurate determination of the degree of cancer development is crucial factor for the treatment.

**Figure 1:**
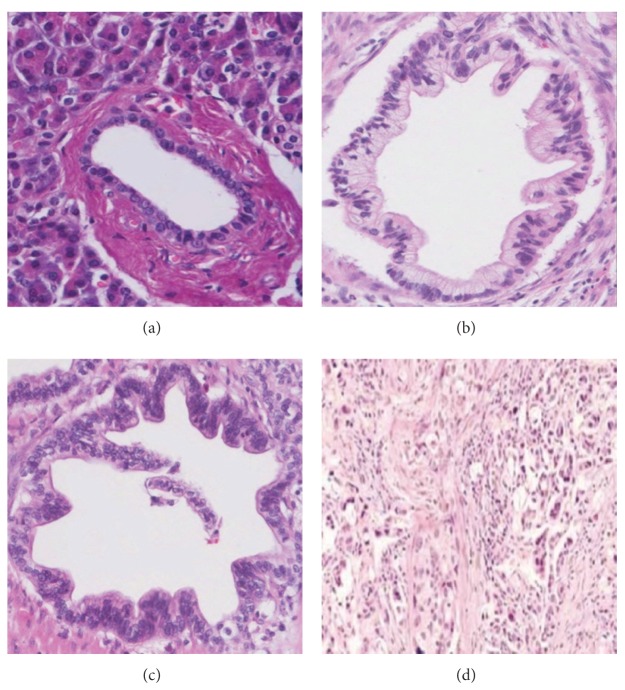
(a) Normal; (b) Grade 1, well differentiated; (c) Grade 2, moderately differentiated; (d) Grade 3, poorly differentiated.

PDAC progression is divided by histological and cytological features and mitotic activity into Grade 1, well-differentiated carcinomas; Grade 2, moderately differentiated carcinomas; and Grade 3, poorly differentiated ductal adenocarcinomas [36, 37]. Grade 1 consists of a duct-like structure combined with medium-sized neoplastic glands. Tubular or cribriform patterns are typical. There may also be small irregular papillary projections without a distinct fibrovascular stalk, particularly in large duct-like structures. Mitotic activity is low. The mucin-producing neoplastic cells tend to be columnar, have eosinophils, and occasionally exhibit pale or even clear cytoplasm. Some neoplastic cell nuclei show loss of polarity. Grade 2 is characterized by a mixture of medium-sized duct-like and tubular structures of variable shapes, embedded in desmoplastic stroma. The duct shape is commonly that of incompletely formed glands. Compared with Grade 1, Grade 2 shows a greater variation in nuclear size, chromatin structure, and prominence of the nucleoli. The cytoplasm is usually slightly eosinophilic, but clear cells are occasionally abundant. Mucin production appears to be decreased, and intraductal *in situ* components are somewhat less frequent than in Grade 1. Grade 3 is infrequent. It is composed of a mixture of densely packed, small, and irregular glands as well as solid tumor cell sheets and nests that entirely replace the acinar tissue. While typical large, duct-like structures and intraductal tumor components are absent, there may be small squamoid features, spindle cells, or anaplastic foci. The neoplastic cells show marked pleomorphism, little or no mucin production, and brisk mitotic activity.  Figure 1 shows Normal, Grade 1, Grade 2, and Grade 3 tissue images.

As described above, Grade 3 is not common. Also, Figure 1 shows that Grade 3 is certainly morphologically different from Grades 1 and 2. Therefore, this paper focuses on the detection of PDAC and the differentiation of Grades 1 and 2.

## 3. System Overview

In this paper, system configuration to diagnose PDAC consists of three phases as follows: segmentation and feature extraction, model learning and validation, and diagnosis. In the first phase, after preprocessing the given tissue image, the image is segmented into three parts. These three parts are the lumen region, epithelial nuclei, and nonepithelial nuclei. Then, according to the characteristics of each part, the features to be used for the classification model are extracted and stored into a feature database. The second phase is the learning and validation of the SVM classification model using the features extracted in the previous step. The final phase carries out PDAC diagnosis for a tissue sample using the generated SVM classification model.

### 3.1. Segmentation for Major Interest Objects

**Figure 2:**
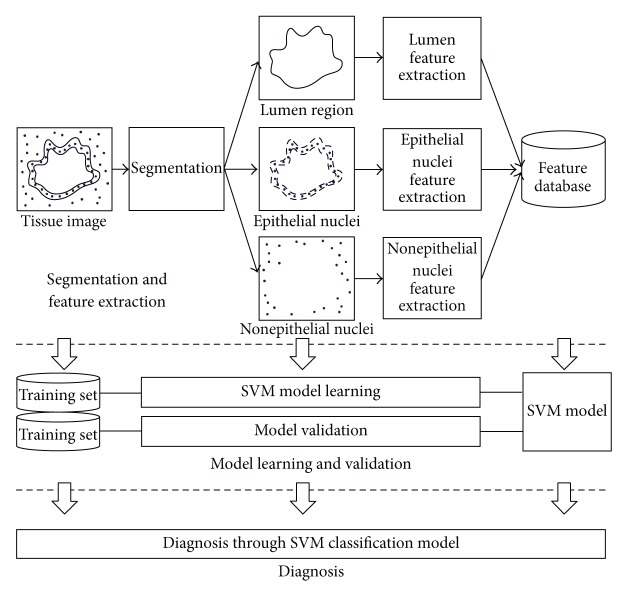
System overview for diagnosing PDAC.

In this section, we describe the method of segmenting three object types in a tissue image. Two of three object types are the lumen and epithelial nucleus constituting a duct. The last one is nonepithelial nucleus.  Figure 2 shows the overall process of identifying three object types from a tissue image.

#### 3.1.1. Lumen Segmentation

**Figure 3:**
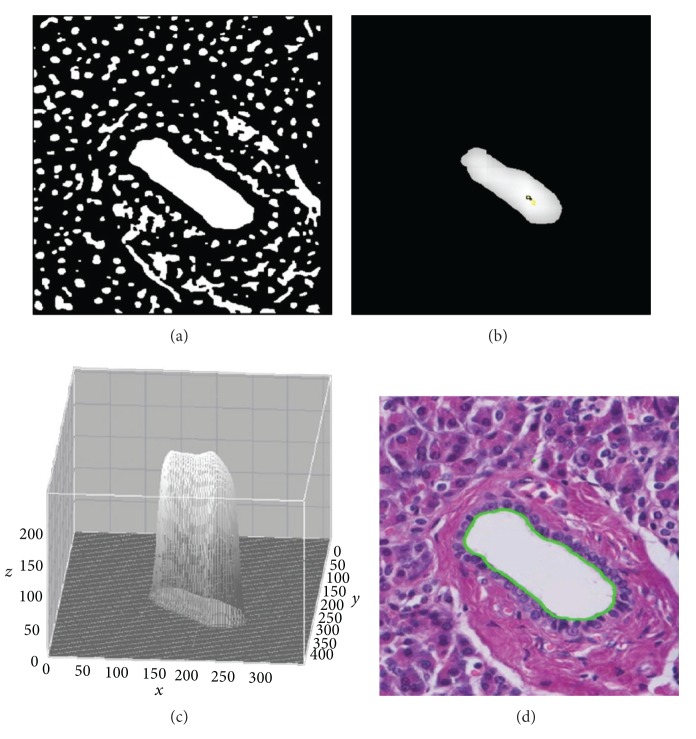
(a) The preprocessed binary image for identifying the lumen boundary; (b) *H*_*T*_(**A**) and candidate seed points (yellow points) for the image; (c) 3D plot for *H*_*T*_(**A**). It is scaled as a range from 0 to 255; (d) the boundary of lumen segmented by candidate seed (green line).

In this paper, the lumen of the tissue image is segmented by a seeded region growing (SRG) algorithm [38]. A beginning point should be designated for the use of the SRG. In the previous research [32], segmentation of the lumen region was automated by identifying candidate seed points within the lumen region. The proposed method is as follows. First of all, in order to facilitate the application of SRG, median filtering algorithm and background correction algorithm [39] are applied on a given image, and then maximum Entropy Threshold [40] is applied to produce a binary image **A**. From the produced binary image **A**, Direction Cumulative Map *H*(**A**) is generated to find seed points. The *H*(**A**) is generated by cumulating only white pixels of four directions (left, right, up, and down) of the binary image **A** and calculating the sum of the square root of the cumulated values. The *H*(**A**) will have higher values around the central area of lumen region. Therefore, the local maximum points of *H*(**A**) would be used as candidate seed points for the SRG algorithm. However, if candidate seed points are generated directly from *H*(**A**), it might generate candidate seed points for unnecessarily narrow areas. As a solution for this problem, *H*_*T*_(**A**) with a threshold of lower value is, instead, employed to acquire candidate seed points. The *Otsu* method [41] is used to determine the threshold. With having the acquired candidate seed points set as a beginning point of parameter, the lumen region can be segmented by SRG algorithm. The boundary of segmented lumen region will be denotated as **B** ^**O**^.  Figure 3 shows the process of segmenting the lumen boundary that has been explained so far.

#### 3.1.2. The Identification of Epithelial Nuclei and Nonepithelial Nuclei

In this phase, we segmented the nuclei of tissue images and separate them into epithelial and nonepithelial nuclei. The process is as follows.

**Figure 4:**
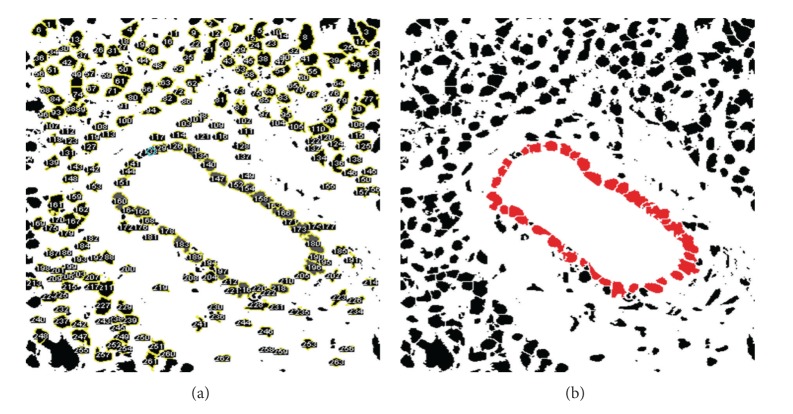
(a) A set of the segmented nuclei, *N*; (b) a set of the epithelial nuclei selected from *N*, *N* ^*E*^ (marked as red).

*(1) Nuclei Segmentation*. This step identifies all nuclei in a tissue image. First, the impurities shown on the tissue image are eliminated by median filter. Then, the color thresholding based on *k*-means [42] removes the parts such as cytoplasm and lumen that are unnecessary to identify nuclei. Next, the holes of the nuclei in the thresholded image are filled with a *hole filling* algorithm [43]. Finally, the nuclei are separated using a *Watershed *algorithm [44]. Then, a set of the segmented nuclei are denoted by *N*.  Figure 4(a) shows the segmented nuclei. 

*(2) Division the Epithelial Nuclei and the Nonepithelial Nuclei*. This step divides the segmented nuclei acquired in the previous step into the epithelial nuclei and the nonepithelial nuclei. Epithelial cells surround the lumen. Therefore, epithelial nuclei are identified by selecting the nearest nuclei to the lumen boundary, **B** ^**O**^, from a set of nuclei *N*. A set of epithelial nuclei is denoted by *N* ^*E*^ and defined as

(1) $$\begin{array}{c} N^{E}={⋃}_{p\in B^{O}}\left\{n|\underset{n\in N}{\text{argmin}}\left(\text{Distnace}\left(\text{Centroid}\left(n\right),p\right)\right)\right\}, \end{array}$$

where *p* is a point in **B** ^**O**^ and the Centroid(·) is a function returning the center point of a given object. The Distance(·,·) is a function returning the Euclidean distance between two given points. Segregation of epithelial nuclei from *N* is a procedure in which the nearest nucleus from each point *p* ∈ **B** ^**O**^ is firstly selected, and the selected nucleus **n** is then included in a set of epithelial nuclei, *N* ^*E*^.  Algorithm 1 presents this procedure.  Figure 4(b) shows the selected epithelial nuclei (marked as red).

Nonepithelial nuclei are acquired by eliminating the identified epithelial nuclei *N* ^*E*^ from a set of nuclei *N* as follows:

(2) $$\begin{array}{c} N^{NE}=N-N^{E}. \end{array}$$

### 3.2. Notations

Table 1 summarizes the notations used in this paper. 

## 4. Proposed Features

A major object examined to diagnose PDAC in a tissue sample is a duct. As described in Section 2, PDAC is classified as Grade 1, 2, or 3 by morphological changes of the duct which is composed of the lumen and the epithelial cells. Therefore, in this section, we propose methods to extract the specific morphological features of the segmented lumen and epithelial nuclei for PDAC diagnosis. 

### 4.1. Lumen Features

In PDAC, a duct seems to have the shape of an incomplete gland with a papillary form and a loss of nucleus polarity. As the processing stage progresses, the shape of the duct becomes more irregular with various atypia. In this subsection, the method representing atypia of a duct and the morphological features for measuring it are discussed.

#### 4.1.1. Representing Atypia of Duct

Generally, a lumen of normal duct seems like a convex hull because atypia rarely appears. Unlike normal formation, as PDAC progresses, atypia of the lumen boundary in the duct becomes more and more irregular. From this standpoint, estimation of ideal lumen boundary of a given duct will be possible and portrayal of atypia of the original lumen will be feasible based on the boundary.

**Figure 5:**
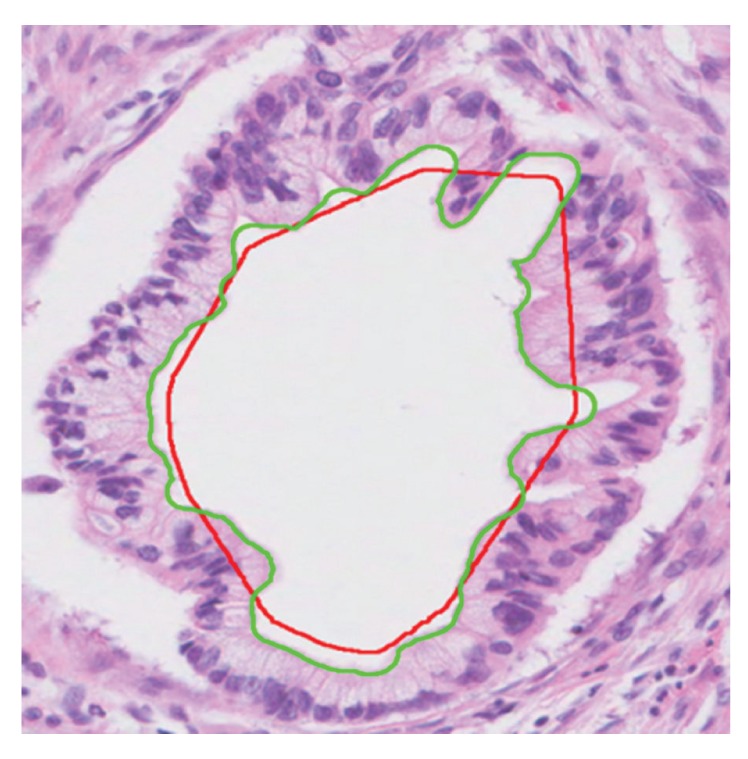
Original lumen boundary **B** ^**O**^ (green line) and ideal lumen boundary **B** ^**I**^ (red line).

*(1)  Ideal Lumen Boundary. *In this step, an original lumen boundary and an estimated ideal lumen boundary are represented as **B** ^**O**^ and **B** ^**I**^, respectively. **B** ^**O**^ and **B** ^**I**^ are, respectively, a sequence of points constituting each lumen boundary. The procedure to estimate the ideal lumen boundary, **B** ^**I**^, is as follows. First, the convex hull, **B** ^**C**^, is obtained from the original lumen boundary **B** ^**O**^. Then, because **B** ^**C**^ is bigger than **B** ^**O**^, the ideal lumen boundary, **B** ^**I**^, is acquired through downsizing **B** ^**C**^ to **B** ^**O**^. The scaling factor, *s*, for scaling **B** ^**C**^ to **B** ^**O**^ is calculated as follows:

(3) $$\begin{array}{c} s=\sqrt{\frac{\text{Area}\left(R^{O}\right)}{\text{Area}\left(R^{C}\right)}}, \end{array}$$

where *R* ^*O*^ represents an area bordering **B** ^**O**^ while *R* ^*C*^ does an area bordering **B** ^**C**^. Area(·) is a function returning area of given region. Therefore, the ideal lumen boundary **B** ^**I**^ is a sequence of points that consist of the boundary of the downsized region by the scaling factor *s* about the center of *R* ^*C*^.  Figure 5 shows the original lumen boundary, **B** ^**O**^, with a green line and the ideal lumen boundary, **B** ^**I**^, with a red line.

**Figure 6:**
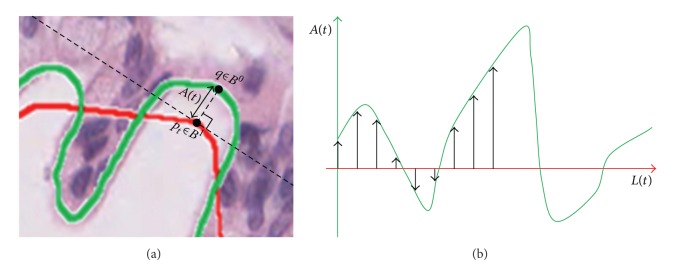
The atypia-amplitude signature with *A*(*t*).

*(2)  Atypia-Amplitude Signature*. In this stage, 1D signature will be proposed as a means to depict atypia of a lumen employing an original lumen boundary and an ideal lumen boundary. The proposed 1D signature visualizes atypia of a lumen by measuring atypia-amplitude between an original lumen boundary and an ideal lumen boundary. The atypia-amplitude is an orthogonal distance with sign between an original lumen boundary and an ideal lumen boundary. It is measured by an atypia-amplitude function *A*(*t*) as follows:

(4) A(t)=sgnt(q)×min⁡pt∈BI,  q∈BO(Distance(pt,q))            such  that  pt⊥q,

where *t* is an index variable, indicating an order of points within an ideal lumen boundary **B** ^**I**^ and sgn⁡_*t*_(*q*) is a function representing sign of a vertical distance between *p*_*t*_ ∈ **B** ^**I**^ and *q* ∈ **B** ^**O**^, which returns +1 or −1, respectively, when a point, *q*, is located either outside or inside the boundary with a point *p*_*t*_ on **B** ^**I**^.  Figure 6(a) shows a process of measuring atypia-amplitude at *p*_*t*_ ∈ **B** ^**I**^. In this example, *A*(*t*) has positive value because *q* is located outside the boundary of *p*_*t*_. 

The 1D signature using the function, *A*(*t*), is named as the atypia-amplitude signature. The atypia-amplitude signature is plotted with points of *x* and *y*. Then *x*-coordinate is the *L*(*t*) meaning the perimeter from *p*_0_ (starting point of **B** ^**I**^) to *p*_*t*_ ∈ **B** ^**I**^, and *y*-coordinate is the *A*(*t*) implying atypia-amplitude at point *p*_*t*_ of **B** ^**I**^. *L*(*t*), the perimeter from *p*_0_ (start point of **B** ^**I**^) to *p*_*t*_, is a sum of the Euclidean distances of each points within the given breadth. The *L*(*t*) is as follows:

(5) $$\begin{array}{c} L\left(t\right)=\sum_{i=1}^{t}\text{Distance}\left(p_{i-1},p_{i}\right),\;\;L\left(0\right)=0. \end{array}$$

Figure 6 shows how to plot the atypia-amplitude signature by *A*(*t*).  Table 2 shows the atypia-amplitudes for Figures 1(a), 1(b), and 1(c). 

#### 4.1.2. Features for Measuring Atypia of Duct

This section introduces features that measure atypia of the lumen quantitatively using atypia-amplitude signature and the ideal lumen boundary developed in the previous phase. The proposed features are RMSAA (root-mean-squared atypia-amplitude), TSAV (total sum of atypia volatilities), AtypiaRatio, and *#*AtypiaRegions (it means the number of atypia regions).

*(1) RMSAA (Root-Mean-Squared Atypia-Amplitude)*. RMSAA is measured by atypia-amplitude signature developed in the previous phase. It is the square root of the mean-squared atypia-amplitude (MSAA). *A*(*t*), the vertical distance between **B** ^**I**^ and **B** ^**O**^, can be interpreted as the residual that represents the difference between the sample value of and the fitted value of the estimated regression model. Likewise, MSAA corresponds to a mean-squared error (MSE) [45] that measures the average squares error of the regression model. RMSAA is defined as follows:

(6) $$\begin{array}{c} \text{RMSAA}=\sqrt{\text{MSAA}}=\sqrt{\frac{1}{m}\sum_{t=1}^{m}A{\left(t\right)}^{2}}. \end{array}$$

Here, *m* is the number of points in **B** ^**I**^. 

**Figure 7:**
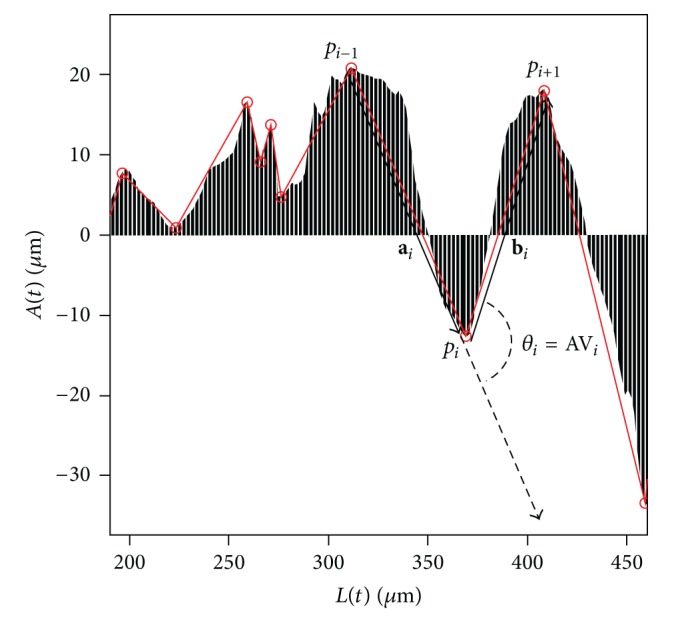
AV_*i*_ at a PIP, *p*_*i*_, is measured as the angle (*θ*_*i*_) between **a**_*i*_ and **b**_*i*_.

*(2) TSAV (Total Sum of Atypia Volatilities).* Variation of the lumen boundary becomes more irregular since the lumen becomes more complex as PDAC develops. TSAV measures the degree of irregularity of the lumen boundary shown in the progress of PDAC. For the calculation of TSAV, major inflection points of atypia-amplitude signature are identified and the sum of diversion at those points is taken into account. In this paper, we use the Perceptual Important Point (PIP) method [46, 47] to find the major inflection points of the atypia-amplitude signature. The PIP method finds critical points that represent important trends of time series data. In this paper, the conventional PIP algorithm that detects a fixed number of PIPs has been modified to find all critical points in the atypia-amplitude signature. The detail about the modified PIP algorithm is included in Appendices A and B. Figure 7 shows a part of atypia-amplitude signature of Grade 2 in Table 2 and PIPs observed by the modified PIP algorithm. 

The TSAV is computed by (7) as the total sum of atypia volatilities (AVs) at PIPs. AV_*i*_ at a PIP, *p*_*i*_, is defined by angle (*θ*_*i*_) between two vectors, **a**_*i*_ = *p*_*i*_ − *p*_*i*−1_ and **b**_*i*_ = *p*_*i*+1_ − *p*_*i*_:

(7) TSAV=∑i=2m−1AVi=∑i=2m−1(arccos(ai·bi||ai||||bi||))(ai=pi−pi−1,bi=pi+1−pi).

Here, *m* is the number of PIPs detected from the atypia-amplitude signature.

**Figure 8:**
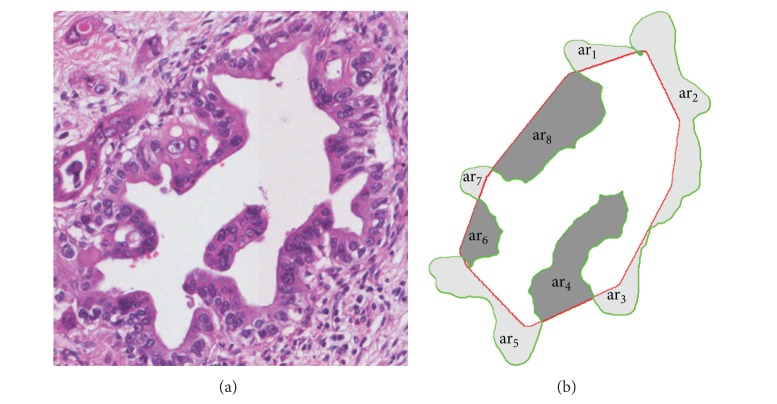
(a) Grade 2 tissue image, (b) atypia regions that are generated by region *R* ^*O*^ and region *R* ^*I*^ of (a).

*(3) Atypia Ratio and the Number of Atypia Regions*. The shape of duct becomes more complex and papillary becomes more vivid as PDAC develops. The original lumen region *R* ^*O*^ of the developed PDAC does not fit into the ideal lumen region, *R* ^*I*^, extending beyond or contracting into the *R* ^*I*^. Thus, in this section, we measure AtypiaRatio and *#*AtypiaRegions (the number of atypia regions) to assess such characteristics. First, a set of atypia regions, **A** **R** = {ar_1_, ar_2_,…, ar_*m*_}, is composed with regions which are generated by separating results of (*R* ^*I*^ ∪ *R* ^*O*^)−(*R* ^*I*^∩*R* ^*O*^) using **B** ^**I**^.  Figure 8 shows the identified atypia regions in Grade 2 tissue image.

The obtained atypia regions are used to come up with AtypiaRatio and *#*AtypiaRegions:

(8) $$\begin{array}{c} \text{AtypiaRatio}=\frac{\sum_{i=1}^{m}\text{Area}\left(\text{a}{\text{r}}_{i}\right)}{\text{Area}\left(R^{O}\right)}\\ \#\text{AtypiaRegions}=\left|\left\{\text{ar}|\text{ar}\in AR,\;\text{Area}\left(\text{ar}\right)>\text{Threshold}\right\}\right|, \end{array}$$

where Area(·) is a function returning the size of a given region. The *m* means the cardinality of the **A** **R**. The AtypiaRatio feature represents the overall degree of distortion within a duct, and the *#*AtypiaRegions feature quantitatively measures the papillary duct by counting the atypia regions. Then, small atypia regions representative of the papillary are excluded from counting by thresholding. The value for thresholding is set 300 *μ*m^2^ by consensus of pathologists at the Pathology Department of Yeongnam University.

### 4.2. Epithelial Cell Feature

Epithelium is another component composing a duct. In most cases, the epithelial cells of PDAC are the mucin-producing neoplastic cells that tend to be columnar, and their nuclei show loss of polarity [34, 48]. In this phase, we introduce the methods of extracting these features from the segmented epithelial nuclei.

**Figure 9:**
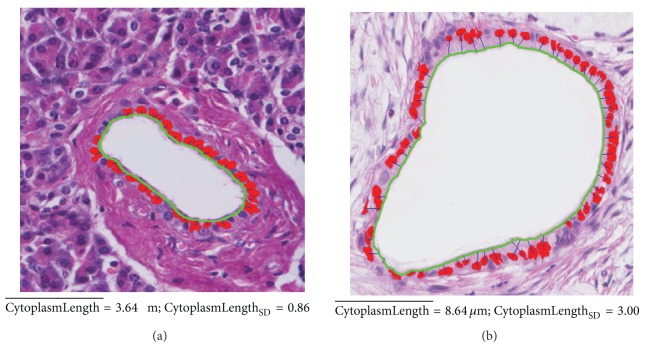
CytoplasmLengths (blue lines) of epithelial cells for Normal and Grade 1.

*(1) Cytoplasm Length*. A duct of normal tissue is surrounded by cube-like epithelial cells. In the PDAC, a duct has columnar epithelium with abundant cytoplasm. The nuclei of columnar cells are oval-shaped. The cytoplasm length of columnar epithelium with abundant cytoplasm is longer than cuboidal epithelium. So, measuring the cytoplasm length of epithelial cells represents whether or not the epithelial cell trend is columnar. We proposed the feature, CytoplasmLength measuring the cytoplasm length of the epithelial cells in [32]. The CytoplasmLength is the orthogonal distance between the epithelial nucleus and the original lumen boundary **B** ^**O**^:

(9) CytoplasmLength(n)=Distance(Centroid(n),q) such  that, Centroid(n)⊥q,

where *q* is a point in **B** ^**O**^ that is orthogonal to Centroid(*n*). Figure 9 shows the measured CytoplasmLengths of Normal and Grade 1. In Figure 9, red regions are epithelial nuclei and green line is the identified lumen boundary. The blue lines between nuclei and lumen boundary are the measured CytoplasmLengths.

*(2) The Standard Deviation of CytoplasmLength*. This feature measures the loss of nuclear polarity that is one of the features of PDAC. The epithelial nuclei with the loss of nuclear polarity have a large deviation between cytoplasm lengths of them because epithelial nuclei are arranged irregularly along the lumen boundary. In contrast, the CytoplasmLengths of normal epithelium have a small deviation because the epithelial nuclei are arranged along the lumen boundary. Therefore, we measure the loss of nuclear polarity by calculating the standard deviation of the CytopalsmLength of epithelial nuclei:

(10) CytoplasmLengthSD=1m∑n∈NE(CytoplasmLength¯−CytoplasmLength(n))2,

where *m* is the cardinality set *N* ^*E*^ and $\bar{\text{CytoplasmLength}}$ is the average of CytoplasmLength.

## 5. Experiments

### 5.1. Image Acquisition and Experimental Environment

We received 21 normal tissue slides and 26 PDAC tissue slides from the Pathology Department of Yeongnam University for our experiments. Those received tissue slides were stained via hematoxylin and eosin. Those tissue slides were scanned into digital slides using the *ScanScope CS System* [49] at 20x magnification. Each digital image of slides is variable depending on acquired tissue.  Table 3 shows the information of digital slide.

In order to assess proposed features, we manually generated images for the experiments from these digital slides to make sure that each includes a duct. Each of the experiment images was formatted into a 24-bit tiff, and their size varied depending on the size of each duct. Important issue in diagnosis is inter- and intraobserver variability leading the diagnosis to be inconsistent, inaccurate, and biased [50]. A similar issue arises from ground truth data of experts for configuration of and performance assessment of a diagnosis system. A feasible way of reducing variability issue of ground truth is to construct ground truth with participation of several experts [51]. This article has three pathologists of the Pathology Department of Yeongnam University participated in an assessment of ground truth. Each image of duct generated from digital slides has been labeled into class with discreet consensus of those three pathologists.  Table 4 presents the number of experiment images labeled by the experts for the assessment of ground truth.

We segmented the given tissue images into three parts (lumen, epithelial nuclei, and nonepithelial nuclei) and extracted existing classical morphological features with the proposed features from each part.  Table 5 shows the features used in the experiments for diagnosing PDAC. The features extracted from each segment are asterisked. 1~12 rows in Table 5 are the existing classical features [5, 24, 39, 52]. 13~18 rows are the proposed features in this paper and our previous study [32]. Because a number of epithelial and nonepithelial cells were found in a captured tissue image, the features of each object were extracted and then averaged to represent features of the tissue image. The experiment environment for feature extraction was performed on a computer with an AMD Athelon II 3 GHZ CPU and 2 G RAM running Windows7 64 bit. The existing and proposed methods for extracting features were implemented by using ImageJ [53], an image processing package based on the JAVA programming language.

### 5.2. Experiment Design

 We compared the performance of the classifiers learned by the classical and proposed features to demonstrate the quality of morphological features that are proposed to diagnose PDAC. In this paper, SVM, a well-founded learning technique based on statistical learning theory [54], was employed as the learning method of the classifier. The SVM shows good generalized performance, because it minimizes the combination of the empirical risk and the VC (Vapnik-Cheronenkis) dimension [55].

The experiment evaluated the classification performance for two cases: classification between Normal and PDAC tissues and classification between Grade 1 and Grade 2 of PDAC. To measure how the proposed features improve the accuracy of classification, the classifiers were learned by feature sets that are configured as existing classical, proposed, and combination features for the three segmented objects (lumen, epithelial nuclei, and nonepithelial nuclei).  Table 6 shows the symbol and dimension for the configured feature sets used in classification experiments. 

Experiment data were generated according to each feature set in Table 6 for the experiments in two cases (Normal versus PDAC and Grade 1 versus Grade 2). Thus, for the experiments to diagnose PDAC in the first case, 13 data sets for 13 feature sets were generated as follows: **D**(CLF), **D**(PLF), **D**(ALF), **D**(CEF), **D**(PEF), **D**(AEF), **D**(CNF), **D**(CDF), **D**(PDF), **D**(ADF), **D**(CTF), **D**(PTF), and **D**(ATF). **D**(·) is the data set that is configured by the given feature set as a parameter. It is denoted as **D**(·) = {(**x**(·)_1_, *y*_1_),…, (**x**(·)_*m*_, *y*_*m*_)}, where **x**(·)_*i*_ is the *i*th feature vector corresponding to a given feature set of parameters (symbols of Table 6) and *y*_*i*_ is its class label. The *y*_*i*_ is either −1, which is Normal, or 1, which is PDAC.

Similarly, for the experiments to grade stages of PDAC in the second case, 13 data sets were generated. In these data sets, *y*_*i*_ = −1 means Grade 1 whereas *y*_*i*_ = 1 means Grade 2. To evaluate the performance of the SVM classifier for each feature set, we configured a training set and test set from the generated data set for the feature set. The ratio of training set to test a set was 60 to 40. In the first experiment (Normal versus PDAC), the experiment data set of PDAC is configured by sampling 80 data from 160 PDAC data of either Grade 1 or Grade 2. Because the number of Normal sample is 80, we limit the number of PDAC for fair evaluation of classifiers.  Table 7 shows the number of training and test data sets used in the experiments of two cases.

In this paper, SVM classifiers used the soft margin method and RBF kernel [54]. Therefore, model parameter *C* and kernel parameter *γ* are required. The optimal classifier parameters (C*, γ*) in which the classification accuracy for 10 cross-validation [56] in the training set is maximized are selected from parameter pairs of (*C*, *γ*)∈{10^−1^, 10^−0.5^,…, 10^4^}×{2^−5^, 2^−4.5^,…, 2^0^} by *Grid Search *[57].

The number of experiment images used in this study is insufficient. Therefore, the classification accuracy of the generated model might be biased [58]. In statistics, to solve this problem, the bootstrap resampling technique [59] is used. We used the bootstrap resampling technique for the unbiased evaluation of classifiers for each feature set. First, we generated 10 training sets and 10 testing sets from data set **D**(·) corresponding to a given feature set for bootstrap evaluation (refer to Table 6). Therefore, the classification performance for each feature set is measured by averaging evaluation results of individually optimized classifiers for 10 training sets and 10 testing data sets. The performance measures used in the experiment are as follows: true positive (TP), true negative (TN), false positive (FP), false negative (FN), sensitivity (SN), Specificity (SP), positive predictive value (PPV), negative predictive value (NPV), and accuracy (ACC). The descriptions for TP, TN, FP, and FN are explained in each experiment. The rest of performance measures are defined as follows:

(11) $$\begin{array}{c} \text{SN}=\frac{\text{TP}}{\text{TP}+\text{FN}},\\ \text{SP}=\frac{\text{TN}}{\text{TN}+\text{FP}},\\ \text{PPV}=\frac{\text{TP}}{\text{TP}+\text{FP}},\\ \text{NPV}=\frac{\text{TN}}{\text{TN}+\text{FN}},\\ \text{ACC}=\frac{\text{TP}+\text{TN}}{\text{TP}+\text{TN}+\text{FP}+\text{FN}}. \end{array}$$

### 5.3. Experimental Results

**Case 1 (Normal versus PDAC)**

**Figure 10:**
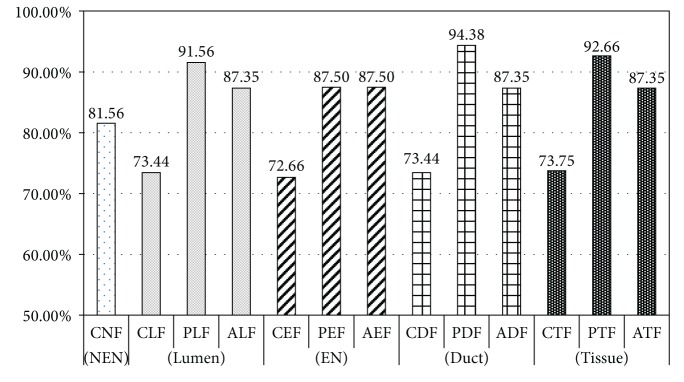
Comparison of classification accuracy for the Normal and PDAC for each feature set.

Table 8 and Figure 10 show the bootstrap evaluation of classifier learned by each feature set, for distinguishing between the Normal and PDAC. The standard deviations of the evaluation results are displayed in parentheses. First, we will compare of classifiers that are learned by feature set extracted from lumen object. In this comparison, the accuracy of classifier learnt by PLF is 91.56% which is about 18% higher than the classification accuracy with CLF (73.44%). The classification accuracy with ALF feature set configured as combination of CLF and PLF has, contrarily, decreased to 87.35%. The results showed that the PLF feature set is more suitable for diagnosing PDAC than classifiers with CLF. However, no improvement in the performance of the classifier with the ALF that is a combination of PLF and CLF was revealed in the results.

Secondly, we will compare classifiers with feature set extracted from epithelial nuclei objects. PEF and AEF show the same accuracy of 87.50%. However, in case of a classifier with AEF, the standard deviation of accuracy was observed as 1.47 which is far stable than PEF of 3.21. Interestingly, AEF is a combination of the PEF and the CEF, its accuracy is dependent on the proposed PEF. Also, the notable point is that the feature dimension of PEF is only two. These results do not only show that the PEF is very suitable for identifying the PDAC but they also prove their effectiveness in diagnostic cost aspect.

In diagnosis of PDAC, a duct that is composed of lumen and epithelium is an important region. Therefore, we thought that the experiment with the combination of features extracted from lumen and epithelial nuclei is of very meaning. For this, we prepared three combination feature sets which include CDF(CLF + CEF), PDF(PLF + PEF), and ADF(CDF + PDF). With these sets, we performed classification. In these experiments, the classification accuracy is improved to 94.38% when the PDF feature set was used. The classification accuracy was measured about 3~7% higher than PLF and AEF (or PEF) that showed best classification performance in each object. It showed that combination of lumen and epithelial nuclei features helps diagnose PDAC.

Consequently, the experimental results of CTF, PTF, and ATF using combination of the feature sets extracted from three objects in a tissue image depended on experimental results conducted in duct object. Thus, it is implied that there are no improvements as a result of combining all features. Further, the experiment with PTF that consists of PDF and CNF showed 2% lower in its accuracy than PDF alone.

**Figure 11:**
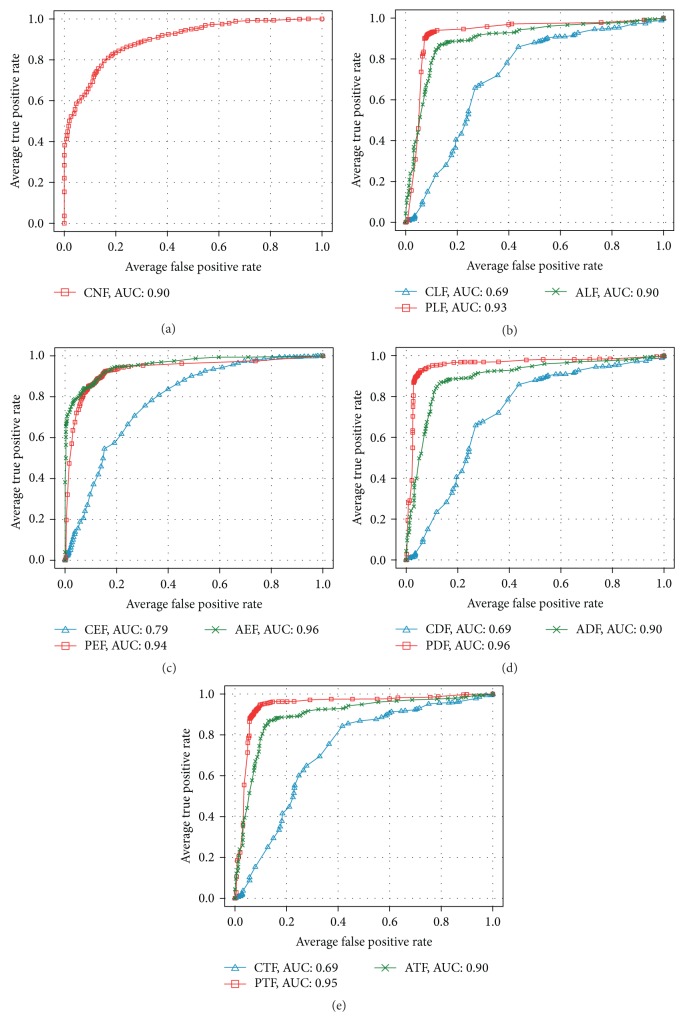
Comparison of ROC curves and AUC values for classifiers in Case 1.

Subsequently, ROC (receiver-operating characteristic) analysis with regard to classifiers learned by each feature set was performed. ROC analysis is being widely used in medical study as a benchmark of accuracy and comparison of diagnosis. ROC analysis examines ROC curves drawn by TP rate (Sensitivity) and FP rate (1-Sensitivity). The examination presents diagnosis accuracy with area under the ROC curve. Swets classified the degree of the accuracy, according to the value of AUC (area under the ROC curve), into noninformative (AUC = 0.5), less accurate (0.5 < AUC ≤ 0.7), moderately accurate (0.7 < AUC ≤ 0.9), highly accurate (0.9 < AUC < 1), and perfect tests (AUC = 1) [60, 61]. In other words, as ROC curve approaches to left hand corner, the accuracy is interpreted as higher.  Figure 11 shows the average ROC graph and value of AUC of 13 classifiers learnt by features of each object.

In the ROC analysis, a classifier learned by PDF displays the highest value of AUC with 0.96. It proves the meaningfulness of combination of epithelial nuclei and lumen features likewise the performance evaluation of the classifier. Subsequent to PDF, a classifier with PEF shows a slightly higher AUC value of 0.94 than that of PLF with 0.93. It is interpreted that classifiers learned by features inclusive of the proposed PDF and PEF show fairly accurate diagnosis with AUC value of above 0.9.

Overall, experiments including proposed feature set show better performance than classifiers with classical feature set. As mentioned in Section 2, the experiments showed that the duct is an important region in diagnosing PDAC. PDF that is composed of PLF and PEF has led to improvement of classifier performance. Also, the classifiers with the proposed PLF and PEF extracted from lumen and epithelial nuclei, respectively, show higher performance than classical feature sets, CLF, CEF, and CNF. It implies that simple morphological features such as Area and Perimeter, are inadequate for finding complicated characteristic of PDAC.

**Case 2 (Grade 1 versus Grade 2)**

**Figure 12:**
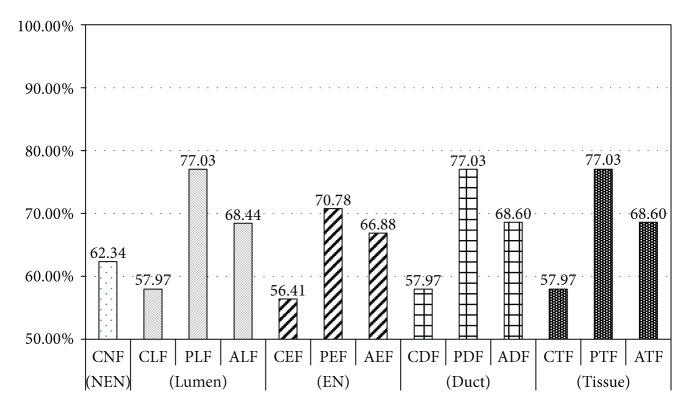
Comparison of classification accuracy for distinguishing between Grade 1 and Grade 2 for each feature set.

In this step, we distinguished between two stages, Grade 1 and Grade 2, of PDAC. As with the experiments in Case 1, we generated 10 training and testing sets for Grade 1 and Grade 2, and we evaluated classification accuracy. The results are shown in Table 9 and Figure 12. The classification results of each feature sets for distinguishing between Grade 1 and Grade 2 show lower classification accuracy than the experiments of distinguishing between Normal and PDAC. Firstly, in the experiments with the lumen object, the classification accuracy with PLF (77.03%) was about 19% higher than that with CLF (57.97%). In particular, specificity of PLF (79.69%) is measured 34% higher than CLF (45.94%), showing performance gain by 73%. In the experiment with ALF, combination of CLF and PLF, the classifier accuracy showed poorer classification performance than the classifier learnt by PLF.

Next, the experiments with the feature sets extracted from epithelial nuclei showed lower accuracy in classification than the experiments with epithelial cells in Case 1. As epithelium cells in all stages of Grade 1 and Grade 2 of PDAC showed columnar and loss of polarity characteristics, distinguishing of stages through them is difficult. Nevertheless, accuracy of the classifier with PEF (70.78%) using only two features increased by about 14% compared to CEF (56.41%). In an experiment using AEF, the accuracy was measured lower than PEF, indicating no performance improvement through the combination of CEF and PEF.

As opposed to the previous experiment that distinguished Normal from PDAC using PDF, an experiment of this case with PDF for classifying stages did not lead to improved performance of a classifier and the evaluation results were the same as experiments with PLF. It proves that the performance enhancement of classifier through PDF, a combination of PLE and PEF, is dependent entirely on PLF while PEF not showing any contribution.

The experiments with combination of feature sets (CTF, PTF, and ATF) extracted from three object types of tissue image showed the same results as those with combination of features sets (CDF, PDF, and ADF) extracted from a duct that consists two object types, lumen and epithelial nucleus.

**Figure 13:**
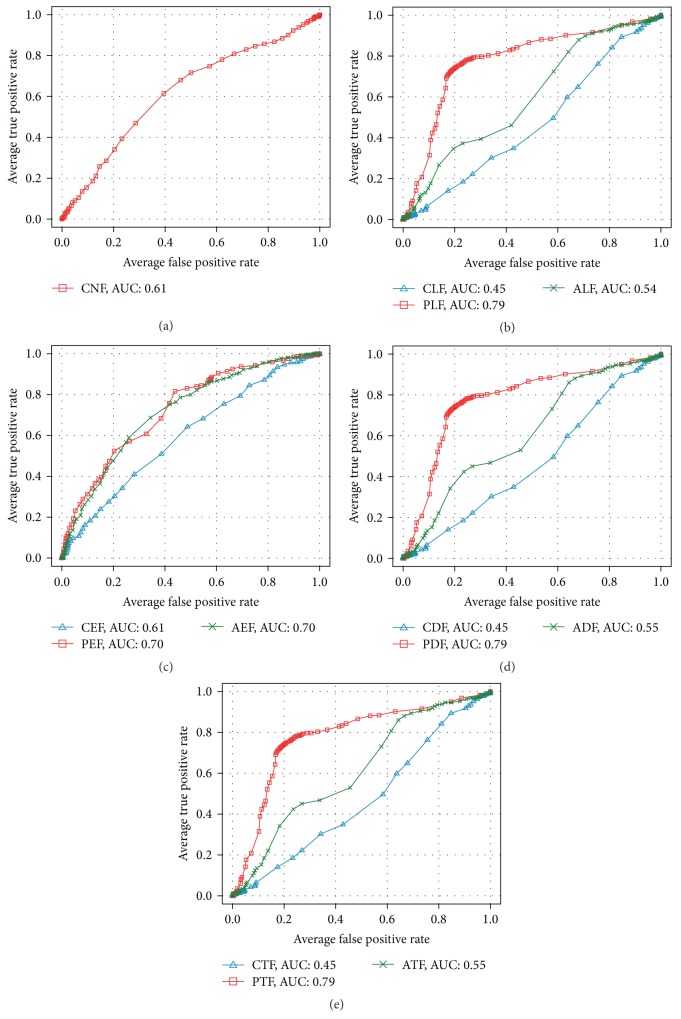
Comparison of ROC curves and AUC values for classifiers in Case 2.

Figure 13 presents ROC graphs and values of AUC averaged by classifiers that distinguishes PDAC stage based on sets of features of each object. In overall, lower performance was detected than Case 1. Classifiers with the classic morphologic features such as CNF, CLF, and CEF provide less accurate diagnosis with AUC values of 0.61, 0.45, and 0.61, respectively. On the contrary, the AUC values of classifiers learned by PLF and PEF are respectively 0.79 and 0.7, showing moderately accurate test results that is one-step higher than AUC values of classifiers learned by existing feature sets such as CEF, CLF, and CNF.

From these experiments of classifying PDAC, classifiers with PLF-contained feature sets show the best performances. As opposed to Case 1 distinguishing Normal from PDAC, no improvements were found with the combination of PEF and PLF. One particular aspect in these experiments is that experiment results of feature sets of lumen object are same as the results of experiments with feature sets of duct object and of tissue object. The feature sets extracted from duct and tissue are composed of the mix of feature sets from lumen object and other objects. It attests that features extracted from lumen are of positive influence to the classifier performance and contain the most information necessary for diagnosing stages of PDAC.

The experiments to classify Grade 1 and Grade 2 in Case 2 showed lower classification performance than the experiments to differentiate between Normal and PDAC in Case 2, both when the proposed features were used and when the existing features were used. This can be explained by the fact that the characteristics of the PDAC commonly appear in Grade 1 and Grade 2 stages. Furthermore, the proposed features lack diagnosing stages of PDAC consisting of similar morphological characteristics since they are designed most of all to distinguish PDAC from Normal tissues. Even if it is so, the proposed feature sets perform better than the classical feature sets.

## 6. Discussion

In this section, we statistically analyzed features that were extracted from the three segmented parts (lumen, epithelial nuclei, and nonepithelial nuclei). Firstly, we assumed that if the extracted features are appropriate for diagnosing PDAC, then the value of features will be different among three populations (Normal, Grade 1, and Grade 2). To statistically show whether the extracted features are different among populations, we performed the ANOVA (ANalysis Of VAriance) for each feature. The null hypothesis for testing if the features are different among populations is as follows:

(12) $$\begin{array}{c} H_{0}:\mu_{N}=\mu_{G1}=\mu_{G2}, \end{array}$$

where *μ*_*N*_, *μ*_*G*1_, and *μ*_*G*2_ are mean population means of Normal, Grade 1, and Grade 2, respectively. The significant test of ANOVA for the features is tested by *F-statistic*. Tables 10, 11, and 12 show *F*-*test* results of null hypothesis (12) for features of each of three object types at the 0.01 level of significance. The *F*-*test* results of features for three object types attest in statistics that there is difference among features in each group (Normal, Grade 1, or Grade 2) in most cases. In *F*-*test* of features extracted from lumen, the existence of disparities was confirmed between features of all groups (Normal, Grade 1, and Grade 2), except for Roundness and Solidity. In *F-test* for features of epithelial nuclei, all features show statistical difference between groups. As for the test of nonepithelial nuclei, MinorAxis and Skewness were only features not showing statistically difference. 

Next, for the post hoc analysis of features that reject the null hypothesis in *F-test*, we performed the multirange test to find whether there are significant differences between means of any population of two. In this paper, the commonly used Fisher's LSD (least significant difference) test was employed for the post hoc analysis of *F*-*test* [62]. In this paper, there are three populations (Normal, Grade 1, and Grade 2) examined. So, LSD-test for total $\left(\begin{array}{c} 3\\ 2 \end{array}\right)$ pairs was performed. The null hypothesis for testing each pair is as follows:

(13) H01:μN=μG1,H02:μN=μG2,H03:μG1=μG2.

The LSD test results features for each of three object types are shown in Tables 13, 14, and 15. The bold values in Tables 13, 14, and 15 mean that the features of all the three hypotheses (13) are rejected in LSD test. Features marked with “-” are those not performed for LSD test since hypothesis test in (12) of *F-test *was rejected. 

Firstly, in LSD test for features extracted from lumen, 12 features except for Circularity and AspectRatio have rejected the three null hypotheses of (13). Of which, 8 features are classical features and 4 features are proposed features. Although there is a number of classical features that have difference between groups, the experiment results using PLF showed more improved performance than experiments using CLF (refer to Tables 8 and 9) in Case 1 and Case 2. 

In epithelial nuclei, 5 features including CytoplasmLength and CytoplasmLength_SD_ rejected the null hypotheses (13). In the case of nonepithelial nuclei, only features of Perimeter, Width, MajorAxis, and Fereter's Diameter rejected the all null hypothesis of (13). The results of LSD test show that null hypothesis, *H*_03_, of (13) to test significant difference between Grade 1 and Grade 2 were not rejected in many features from two distinct object types, epithelial nucleus and nonepithelial nucleus. 

In LSD test of lumen features, features that rejected null hypothesis (12) of *F-test* but not in *H*_03_ (13) are only two, Circularity and AspectRatio. However, in LSD tests of epithelial nucleus features, only 6 features of 14 features that rejected null hypothesis (12) of *F-test* reject *H*_03_. In LSD-test of nonepithelial nuclei, only 4 features rejected *H*_03_. Through LSD test, it has been confirmed that the lumen is the most important object in the diagnosis of PDAC and its stages. Furthermore, LSD test results describe the reason why classification performance of the experiments in Case 2 (Grade 1 versus Grade 2) is lower than that of them in Case 1 (Normal versus PDAC). 

## 7. Conclusions

This paper proposed features to diagnose PDAC and to identify the stages of PDAC. PDAC is mainly diagnosed by investigating a duct that consists of lumen and epithelial cells. We segmented a tissue image into three parts: lumen, epithelial nuclei, and nonepithelial nuclei. Then, we proposed methods for extracting new morphological features from the epithelial cells and lumen parts that are segmented. In PDAC, the shape of the duct is more complex than Normal. Thus, this paper proposed the features for measuring atypia of the duct based on this perspective. We transformed the lumen into the atypia-amplitude signature with the atypia-amplitude function *A*(*t*) to intuitively represent the variation of a duct and proposed RMSAA for measuring the deviation of the aytpia-amplitudes and TSAV for measuring the volatility at PIP points of it. And, using the ideal lumen and original lumen regions, we measured AtypiaRatio, which represents the overall degree of distortion of a duct and #AtypiaRegions that quantify the papillary ducts. Also, we used features such as CytoplasmLength and CytoplasmLength_SD_ to quantitatively measure the morphological features from segmented epithelial nuclei. The experiments' results show that the proposed features are suitable to diagnose PDAC and to distinguish between the two stages, Grade 1 and Grade 2, of PDAC.

## Figures and Tables

**Figure 14:**
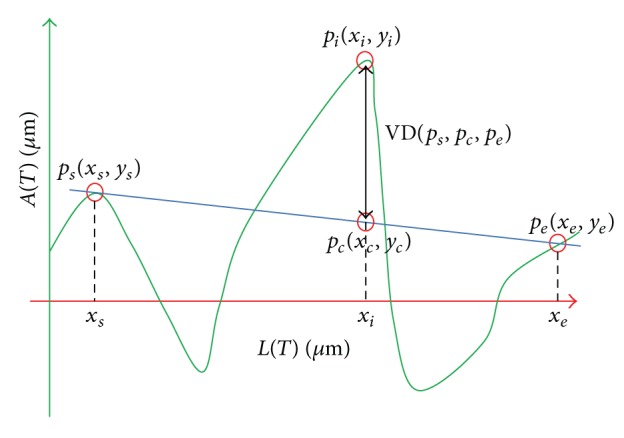
VD(*p*_*s*_, *p*_*i*_, *p*_*e*_) at *p*_*i*_ between PIPs, *p*_*s*_ and *p*_*e*_.

**Figure 15:**
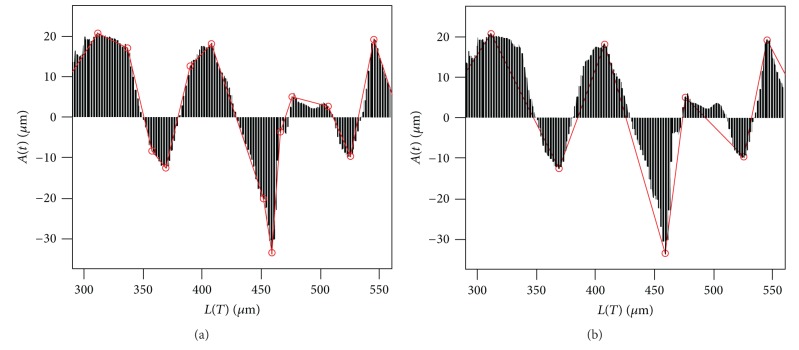
(a) PIPs identified by Algorithm 2; (b) PIPs after applying Algorithm 5 to (a) (red circles represent detected PIPs).

**Algorithm 1:**
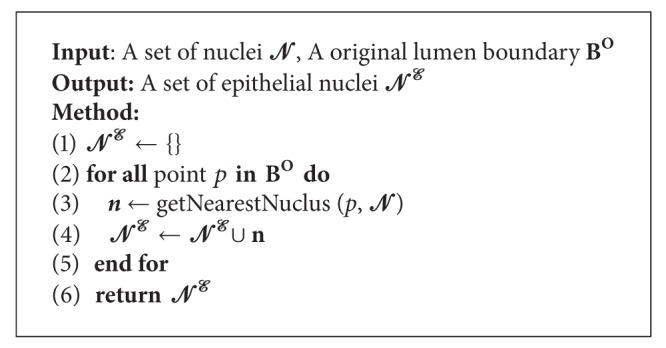
Selection_Epithelial_Nuclei (*N*).

**Algorithm 2:**
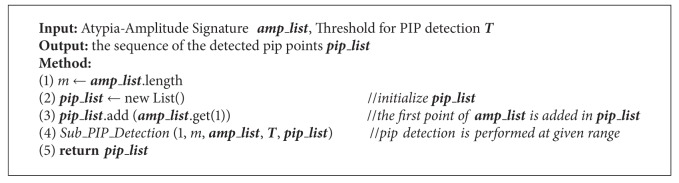
*PIP_Detection_For_Atypia_ Amplitude_Signature*  (***amp_list***, ***T***).

**Algorithm 3:**
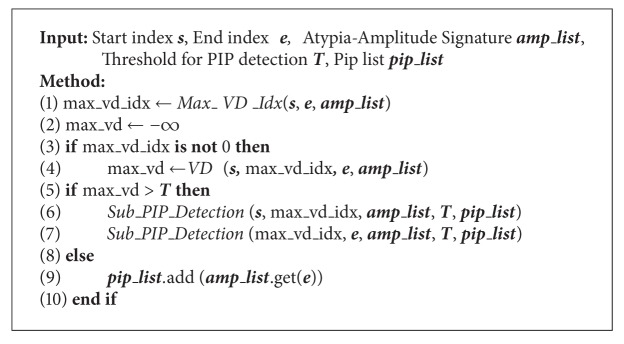
*Sub_PIP_Detection*  (***s***, ***e***, ***amp_list***, ***T***, *pip_list*)*. *

**Algorithm 4:**
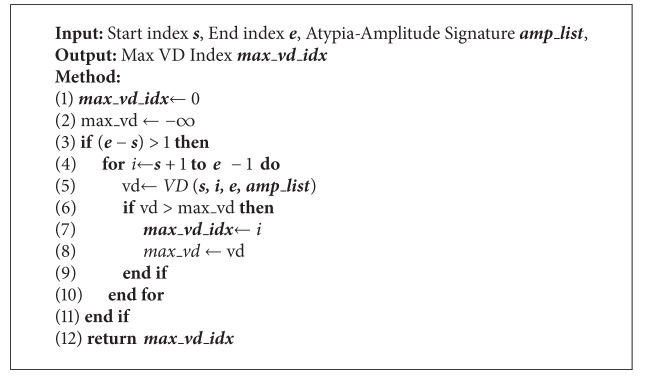
*Max_ VD _Idx*  (***s***, ***e***, ***amp_list***).

**Algorithm 5:**
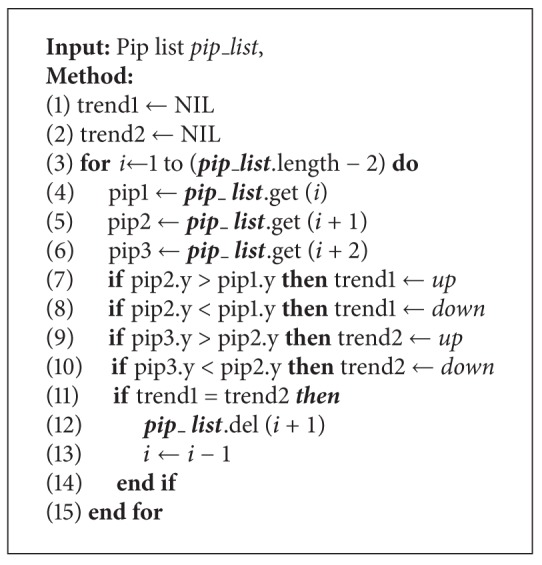
*Post_Porocessing_PIPs*  (***pip_list***)*. *

**Table 1:** List of notations.

Symbol	Description
*p *	The *p* is a pair of numbers that are *x* and *y* coordinates *p* = (*x*, *y*)
**A**	The matrix representation for preprocessed binary image
*H*(**A**)	The direction cumulative histogram for **A**
*H*_*T*_(**A**)	The thresholded *H*(**A**)
**B** ^**O**^	A sequence of points that consist of the original lumen boundary
**B** ^**C**^	A sequence of points for the convex hull of **B** ^**O**^
**B** ^**I**^	A sequence of points for the ideal lumen boundary that is estimated from **B** ^**O**^
*N*	A set of nuclei *N* = {*n*_1_, *n*_2_,…, *n*_*m*_}
*N* ^*E*^	A set of epithelial nuclei *N* ^*E*^ ⊂ *N*
*N* ^*NE*^	A set of nonepithelial nuclei *N* ^*NE*^ = *N* − *N* ^*E*^
Distance(·,·)	The Euclidean distance function
Centroid(·)	The function returning center point of given object
*A*(*t*)	The atypia-amplitude function
*L*(*t*)	The perimeter from *p*_0_ (start point) to *p*_*t*_ (*t*th point)
**A** **R**	A set of atypia regions **A** **R** *** *** = {ar_1_, ar_2_,…, ar_*m*_}
*R* ^*O*^	The region surrounded by **B** ^**O**^
*R* ^*C*^	The region surrounded by **B** ^**C**^
*R* ^*I*^	The region surrounded by **B** ^**I**^
Area (·)	The function returning area of given region
**D**(·)	The data set for given feature set **D**(·) = {(**x**_1_(·),*y*_1_),…, (**x**_*m*_(·),*y*_*m*_)}, **x**(·) is feature vector for given feature set and *y*_*i*_ is class label for **x**(·)

**Table 2:** The atypia-amplitude signature for Figures 1(a), 1(b), and 1(c).

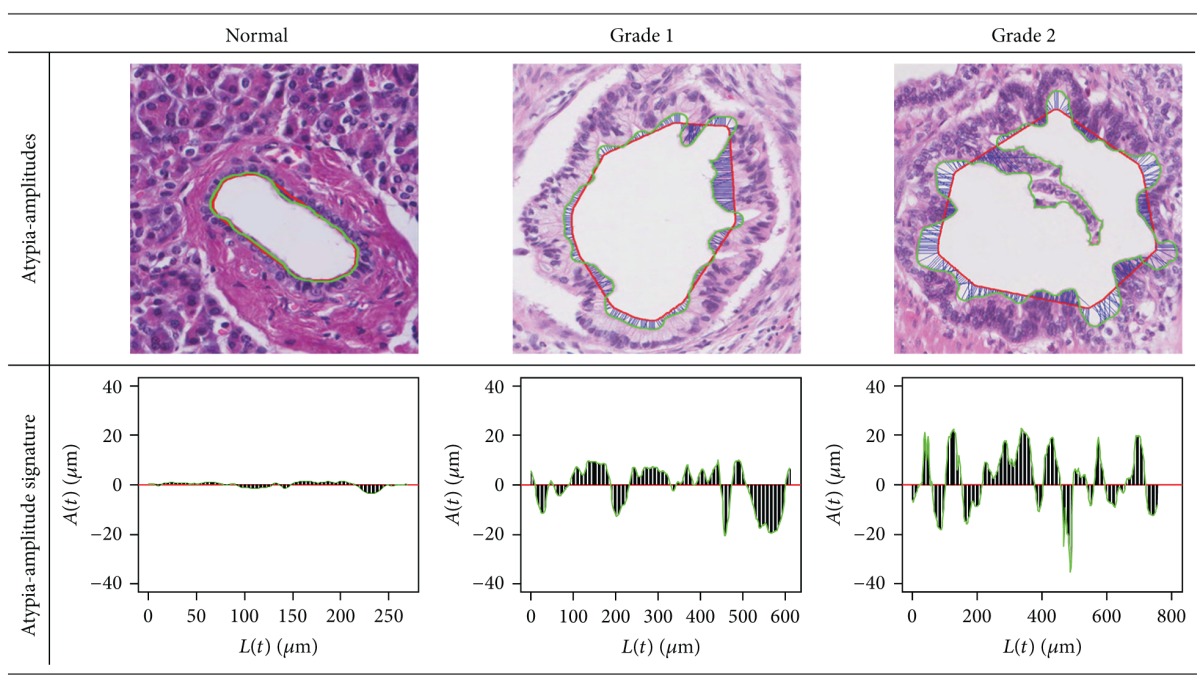

**Table 3:** Information of the digital slide.

Digital slide size	Variable size
Image resolution	0.492 *μ*m/pixel
Image type	SVS/JPEG2000
Image channels	3
Image bit depth	8 bits
Magnification	20x
Organization	Tiled
Tile width	240 pixels
Tile height	240 pixels

**Table 4:** The obtained experimental images.

Type	Number of images
Normal	80
Grade 1	80
Grade 2	80

**Table 5:** Morphological features used in the classification experiment.

No.	Feature	Description	NEN^1^	Lumen	EN^2^
1	Area	Area of selection in square pixels	*	*	*
2	Perimeter	The length of the outside boundary of the selection	*	*	*
3	Width	Width of the smallest rectangle enclosing the selection	*	*	*
4	Height	Height of the smallest rectangle enclosing the selection	*	*	*
5	MajorAxis	Major (primary) axis length of the best fitting ellipse	*	*	*
6	MinorAxis	Minor (secondary) axis length of the best fitting ellipse	*	*	*
7	Circularity	4*π* × (Area/Perimeter^2^). A value of 1.0 indicates a perfect circle	*	*	*
8	Feret's diameter	The longest distance between any two points along the selection boundary	*	*	*
9	AspectRatio	MajorAxis/MinorAxis	*	*	*
10	Skewness	The third order moment about the mean	*	*	*
11	Roundness	4 × Area/(*π* × MajorAxis^2^). The measure of the sharpness of particle's edge and corners	*	*	*
12	Solidity	Area/ConvexArea	*	*	*
13	RMSAA	Root-mean-squared atypia-amplitude		*	
14	TSAV	Total sum of atypia volatilities for PIPs		*	
15	AtypiaRatio	The ratio of atypia region		*	
16	#AtypiaRegions	The number of atypia regions for identifying papillary		*	
17	CytoplasmLength	The cytoplasm length of epithelial nucleus [32]			*
18	CytoplasmLength_SD_	The standard deviation of CytoplasmLength			*

NEN^1^: nonepithelial nuclei; EN^2^: epithelial nuclei.

**Table 6:** The symbol and the dimension of feature sets that are configured by the object features and combined features.

Object	Symbol	Feature set	Dimension
NEN	CNF	Classical nonepithelial nuclei features	12

Lumen	CLF	Classical lumen features	12
	PLF	Proposed lumen features	4
	ALF	CLF + PLF	16

EN	CEF	Classical epithelial nuclei features	12
	PEF	Proposed epithelial nuclei features	2
	AEF	CEF + PEF	14

Duct	CDF	Classical duct features (CLF + CEF)	24
	PDF	Proposed duct features (PLF + PEF)	6
	ADF	CDF + PDF	30

Tissue	CTF	Classical features extracted from three objects (CLF + CEF + CNF)	36
	PTF	Proposed features extracted from three objects (PLF + PEF + CNF)	18
	ATF	All features (CLF + CEF + PLF + PEF + CNF)	42

**Table 7:** The number of training and testing data sets for learning and evaluating the SVM classifier.

Experiment	Class	Number of training data	Number of testing data

Case 1: Normal versus PDAC	Normal	48	32
	PDAC	48	32

Case 2: Grade 1 versus Grade 2	Grade 1	48	32
	Grade 2	48	32

**Table 8:** Evaluation results for distinguishing Normal and PDAC to each feature set.

Object	Feature set	TN	FP	FN	TP	SN (%)	SP (%)	PPV (%)	NPV (%)	ACC (%)
NEN	CNF	26.50	6.30	5.50	25.70	82.82	80.32	81.07	82.50	81.56
		(1.58)	(2.11)	(1.58)	(2.11)	(4.94)	(6.60)	(5.17)	(4.22)	(3.95)

	CLF	27.30	12.30	4.70	19.70	85.32	61.57	69.38	81.72	73.44
		(2.21)	(3.27)	(2.21)	(3.27)	(6.92)	(10.21)	(4.22)	(6.25)	(3.13)
Lumen	PLF	29.30	2.70	2.70	29.30	91.57	91.57	91.99	91.88	91.56
		(1.77)	(2.00)	(1.77)	(2.00)	(5.52)	(6.26)	(5.45)	(4.70)	(3.14)
	ALF	27.50	3.60	4.50	28.40	85.94	88.75	88.96	86.36	87.35
		(0.85)	(2.46)	(0.85)	(2.46)	(2.66)	(7.68)	(6.92)	(2.12)	(3.57)

	CEF	25.10	10.60	6.90	21.40	78.44	66.88	70.72	75.75	72.66
		(1.66)	(2.84)	(1.66)	(2.84)	(5.20)	(8.86)	(4.97)	(3.99)	(3.84)
EN	PEF	27.50	3.50	4.50	28.50	85.94	89.07	89.52	86.67	87.50
		(1.51)	(2.95)	(1.51)	(2.95)	(4.72)	(9.23)	(6.80)	(3.25)	(3.21)
	AEF	27.00	3.00	5.00	29.00	84.38	90.63	90.55	85.73	87.50
		(2.05)	(1.89)	(2.05)	(1.89)	(6.42)	(5.89)	(5.01)	(4.53)	(1.47)

	CDF	27.30	12.30	4.70	19.70	85.32	61.57	69.38	81.72	73.44
		(2.21)	(3.27)	(2.21)	(3.27)	(6.92)	(10.21)	(4.22)	(6.25)	(3.13)
Duct	PDF	29.80	1.40	2.20	30.60	93.13	95.63	95.78	93.50	94.38
		(1.40)	(1.51)	(1.40)	(1.51)	(4.37)	(4.71)	(4.34)	(3.80)	(2.35)
	ADF	27.50	3.60	4.50	28.40	85.94	88.75	88.96	86.36	87.35
		(0.85)	(2.46)	(0.85)	(2.46)	(2.66)	(7.68)	(6.92)	(2.12)	(3.57)

	CTF	26.90	11.70	5.10	20.30	84.06	63.44	70.34	80.92	73.75
		(2.28)	(3.74)	(2.28)	(3.74)	(7.13)	(11.70)	(5.32)	(6.11)	(3.44)
Tissue	PTF	29.80	2.50	2.20	29.50	93.13	92.19	92.74	93.26	92.66
		(1.32)	(2.22)	(1.32)	(2.22)	(4.11)	(6.95)	(6.15)	(3.76)	(3.21)
	ATF	27.50	3.60	4.50	28.40	85.94	88.75	88.96	86.36	87.35
		(0.85)	(2.46)	(0.85)	(2.46)	(2.66)	(7.68)	(6.92)	(2.12)	(3.57)

TP (true positive): the number of PDACs that are correctly classified as PDACs.

FP (false positive): the number of Normals that are incorrectly classified as PDACs.

FN (false negative): the number of PDACs that are incorrectly classified as Normals.

TN (true negative): the number of Normals that are correctly classified as Normals.

**Table 9:** Evaluation results for distinguishing between Grade 1 and Grade 2 to each feature set.

Object	Feature set	TN	FP	FN	TP	SN (%)	SP (%)	PPV (%)	NPV (%)	ACC (%)
NEN	CNF	19.60	11.70	12.40	20.30	61.25	63.44	64.35	63.29	62.34
		(6.26)	(4.97)	(6.26)	(4.97)	(19.56)	(15.52)	(8.93)	(9.49)	(7.78)

	CLF	22.40	17.30	9.60	14.70	70.00	45.94	58.39	66.13	57.97
		(5.99)	(6.95)	(5.99)	(6.95)	(18.70)	(21.70)	(10.63)	(16.21)	(5.23)
Lumen	PLF	23.80	6.50	8.20	25.50	74.38	79.69	79.65	75.40	77.03
		(1.32)	(3.95)	(1.32)	(3.95)	(4.11)	(12.35)	(10.15)	(4.96)	(6.83)
	ALF	25.20	13.40	6.80	18.60	78.75	58.13	67.05	73.46	68.44
		(2.90)	(6.31)	(2.90)	(6.31)	(9.06)	(19.72)	(9.88)	(5.85)	(7.39)

	CEF	18.60	14.50	13.40	17.50	58.13	54.69	56.45	57.03	56.41
		(4.30)	(4.14)	(4.30)	(4.14)	(13.44)	(12.95)	(7.17)	(9.10)	(7.53)
EN	PEF	22.20	8.90	9.80	23.10	69.38	72.19	71.89	70.04	70.78
		(1.14)	(2.92)	(1.14)	(2.92)	(3.55)	(9.14)	(6.94)	(4.08)	(5.26)
	AEF	22.40	11.60	9.60	20.40	70.00	63.75	65.95	68.16	66.88
		(2.32)	(2.27)	(2.32)	(2.27)	(7.25)	(7.10)	(5.42)	(5.84)	(5.50)

	CDF	22.40	17.30	9.60	14.70	70.00	45.94	58.39	66.13	57.97
		(5.99)	(6.95)	(5.99)	(6.95)	(18.70)	(21.70)	(10.63)	(16.21)	(5.23)
Duct	PDF	23.80	6.50	8.20	25.50	74.38	79.69	79.65	75.40	77.03
		(1.32)	(3.95)	(1.32)	(3.95)	(4.11)	(12.35)	(10.15)	(4.96)	(6.83)
	ADF	25.10	13.20	6.90	18.80	78.44	58.75	67.25	73.40	68.60
		(2.88)	(6.21)	(2.88)	(6.21)	(9.02)	(19.42)	(9.75)	(5.88)	(7.31)

	CTF	22.40	17.30	9.60	14.70	70.00	45.94	58.39	66.13	57.97
		(5.99)	(6.95)	(5.99)	(6.95)	(18.70)	(21.70)	(10.63)	(16.21)	(5.23)
Tissue	PTF	23.80	6.50	8.20	25.50	74.38	79.69	79.65	75.40	77.03
		(1.32)	(3.95)	(1.32)	(3.95)	(4.11)	(12.35)	(10.15)	(4.96)	(6.83)
	ATF	25.10	13.20	6.90	18.80	78.44	58.75	67.25	73.40	68.60
		(2.88)	(6.21)	(2.88)	(6.21)	(9.02)	(19.42)	(9.75)	(5.88)	(7.31)

TP (true positive): the number of Grade 2s that are correctly classified as Grade 2s.

FP (false positive): the number of Grade 1s that are incorrectly classified as Grade 2s.

FN (false negative): the number of Grade 2s that are incorrectly classified as Grade 1s.

TN (true negative): the number of Grade 1s that are correctly classified as Grade 1s.

**Table 10:** The statistics for nonepithelial nuclei features and the results of *F-*test.

Lumen features	Normal (df = 79)	Grade 1 (df = 79)	Grade 2 (df = 79)	*F*-tests (*μ*_*N*_ = *μ*_*G*1_ = *μ*_*G*2_)
	$\bar{N}$ (C.I^a^)	$\bar{G1}$ (C.I)	$\bar{G2}$ (C.I)	*F*-value

Area (*μ*m^2^)	3.68*E* + 01 (±1.57*E* + 00)	4.09*E* + 01 (±1.67*E* + 00)	3.84*E* + 01 (±1.57*E* + 00)	1.13*E* + 01*
Perimeter (*μ*m)	2.66*E* + 01 (±6.35*E* − 01)	2.95*E* + 01 (±7.09*E* − 01)	2.84*E* + 01 (±6.67*E* − 01)	3.30*E* + 01*
Width (*μ*m)	7.50*E* + 00 (±1.85*E* − 01)	8.48*E* + 00 (±2.34*E* − 01)	7.97*E* + 00 (±2.18*E* − 01)	3.64*E* + 01*
Height (*μ*m)	7.92*E* + 00 (±2.20*E* − 01)	8.46*E* + 00 (±2.57*E* − 01)	8.31*E* + 00 (±2.41*E* − 01)	9.47*E* + 00*
MajorAxis (*μ*m)	8.58*E* + 00 (±1.76*E* − 01)	9.68*E* + 00 (±1.78*E* − 01)	9.28*E* + 00 (±1.83*E* − 01)	6.78*E* + 01*
MinorAxis (*μ*m)	5.37*E* + 00 (±1.20*E* − 01)	5.28*E* + 00 (±1.35*E* − 01)	5.17*E* + 00 (±1.28*E* − 01)	4.17*E* + 00
Circularity	6.67*E* − 01 (±1.08*E* − 02)	6.01*E* − 01 (±1.17*E* − 02)	6.10*E* − 01 (±1.24*E* − 02)	6.63*E* + 01*
Feret's diameter (*μ*m)	9.63*E* + 00 (±2.02*E* − 01)	1.09*E* + 01 (±2.28*E* − 01)	1.04*E* + 01 (±2.12*E* − 01)	5.80*E* + 01*
Skewness	3.35*E* − 01 (±1.03*E* − 01)	4.23*E* − 01 (±2.02*E* − 01)	4.00*E* − 01 (±1.52*E* − 01)	5.85*E* − 01
AspectRatio	1.66*E* + 00 (±3.58*E* − 02)	1.98*E* + 00 (±6.07*E* − 02)	1.91*E* + 00 (±5.62*E* − 02)	7.55*E* + 01*
Roundness	6.50*E* − 01 (±1.14*E* − 02)	5.73*E* − 01 (±1.25*E* − 02)	5.84*E* − 01 (±1.21*E* − 02)	8.30*E* + 01*
Solidity	8.38*E* − 01 (±4.40*E* − 03)	8.19*E* − 01 (±4.58*E* − 03)	8.19*E* − 01 (±4.81*E* − 03)	3.96*E* + 01*

C.I^a^: confidence interval.

*It indicates features whose null hypothesis was rejected with *F-*value > *F*_0.01_(2,237).

**Table 11:** The statistics for lumen features and the results of *F-*test.

Lumen Features	Normal (df = 79)	Grade 1 (df = 79)	Grade 2 (df = 79)	*F*-tests (*μ*_*N*_ = *μ*_*G*1_ = *μ*_*G*2_)
	$\bar{N}$(C.I)	$\bar{G1}$(C.I)	$\bar{G2}$(C.I)	*F*-value
Area (*μ*m^2^)	3.78*E* + 04 (±9.35*E* + 03)	6.77*E* + 04 (±1.23*E* + 04)	1.19*E* + 05 (2.54*E* + 04)	4.00*E* + 01*
Perimeter (*μ*m)	1.49*E* + 03 (±1.78*E* + 02)	2.09*E* + 03 (±1.74*E* + 02)	2.73*E* + 03 (2.69*E* + 02)	6.05*E* + 01*
Width (*μ*m)	1.86*E* + 02 (±2.64*E* + 01)	2.73*E* + 02 (±2.93*E* + 01)	3.66*E* + 02 (4.70*E* + 01)	4.48*E* + 01*
Height (*μ*m)	1.79*E* + 02 (±1.99*E* + 01)	2.39*E* + 02 (±2.31*E* + 01)	3.05*E* + 02 (2.78*E* + 01)	4.88*E* + 01*
MajorAxis (*μ*m)	2.29*E* + 02 (±2.94*E* + 01)	3.36*E* + 02 (±3.23*E* + 01)	4.36*E* + 02 (5.01*E* + 01)	5.10*E* + 01*
MinorAxis (*μ*m)	1.83*E* + 02 (±2.09*E* + 01)	2.42*E* + 02 (±2.01*E* + 01)	3.20*E* + 02 (2.99*E* + 01)	5.70*E* + 01*
Circularity	1.86*E* − 01 (±1.10*E* − 03)	1.82*E* − 01 (±2.70*E* − 03)	1.83*E* − 01 (2.59*E* − 03)	5.91*E* + 00*
Feret's diameter (*μ*m)	5.31*E* + 02 (±6.42*E* + 01)	7.53*E* + 02 (±6.43*E* + 01)	9.84*E* + 02 (1.00*E* + 02)	5.87*E* + 01*
Skewness	2.15*E* − 01 (±1.71*E* − 01)	−7.27*E* − 01 (±1.91*E* − 01)	−9.88*E* − 01 (1.71*E* − 01)	8.79*E* + 01*
AspectRatio	1.25*E* + 00 (±5.58*E* − 02)	1.41*E* + 00 (±1.12*E* − 01)	1.38*E* + 00 (1.10*E* − 01)	5.68*E* + 00*
Roundness	8.15*E* − 01 (±3.22*E* − 02)	7.47*E* − 01 (±4.62*E* − 02)	7.60*E* − 01 (4.53*E* − 02)	5.21*E* + 00
Solidity	1.00*E* + 00 (±0.00*E* + 00)	1.00*E* + 00 (±0.00*E* + 00)	1.00*E* + 00 (0.00*E* + 00)	1.00*E* + 00
AtypiaRatio	5.11*E* − 02 (±1.09*E* − 02)	2.72*E* − 01 (±6.89*E* − 02)	4.69*E* − 01 (7.09*E* − 02)	9.22*E* + 01*
#AtypiaRegions	7.50*E* − 02 (±1.39*E* − 01)	2.48*E* + 00 (±5.56*E* − 01)	4.84*E* + 00 (6.39*E* − 01)	1.61*E* + 02*
RMSAA	1.03*E* + 00 (±2.60*E* − 01)	8.85*E* + 00 (±2.97*E* + 00)	1.88*E* + 01 (3.63*E* + 00)	7.51*E* + 01*
TSAV (rad)	1.95*E* + 02 (±1.88*E* + 02)	8.65*E* + 03 (±3.45*E* + 03)	2.61*E* + 04 (6.45*E* + 03)	6.80*E* + 01*

*It indicates features whose null hypothesis was rejected with *F-*value > *F*_0.01_(2,237).

**Table 12:** The statistics for the epithelial nuclei features and the results of *F-*test.

Lumen features	Normal (df = 79)	Grade 1 (df = 79)	Grade 2 (df = 79)	*F*-tests (*μ*_*N*_ = *μ*_*G*1_ = *μ*_*G*2_)
	$\bar{N}$ (C.I)	$\bar{G1}$ (C.I)	$\bar{G2}$ (C.I)	*F*-value
Area (*μ*m^2^)	4.22*E* + 01 (±2.67*E* + 00)	5.26*E* + 01 (±2.82*E* + 00)	4.83*E* + 01 (±2.94*E* + 00)	2.43*E* + 01*
Perimeter (*μ*m)	2.81*E* + 01 (±1.09*E* + 00)	3.20*E* + 01 (±9.63*E* − 01)	3.10*E* + 01 (±9.88*E* − 01)	2.81*E* + 01*
Width (*μ*m)	8.04*E* + 00 (±3.11*E* − 01)	9.11*E* + 00 (±2.51*E* − 01)	8.65*E* + 00 (±2.72*E* − 01)	2.58*E* + 01*
Height (*μ*m)	8.16*E* + 00 (±3.38*E* − 01)	9.19*E* + 00 (±3.17*E* − 01)	8.92*E* + 00 (±2.59*E* − 01)	2.10*E* + 01*
MajorAxis (*μ*m)	8.87*E* + 00 (±2.99*E* − 01)	1.01*E* + 01 (±2.55*E* − 01)	9.66*E* + 00 (±2.42*E* − 01)	3.73*E* + 01*
MinorAxis (*μ*m)	5.92*E* + 00 (±2.01*E* − 01)	6.45*E* + 00 (±2.10*E* − 01)	6.13*E* + 00 (±2.25*E* − 01)	1.10*E* + 01*
Circularity	6.87*E* − 01 (±1.95*E* − 02)	6.47*E* − 01 (±1.69*E* − 02)	6.33*E* − 01 (±1.42*E* − 02)	1.86*E* + 01*
Feret's diameter (*μ*m)	9.94*E* + 00 (±3.52*E* − 01)	1.13*E* + 01 (±2.97*E* − 01)	1.09*E* + 01 (±2.83*E* − 01)	3.46*E* + 01*
Skewness	3.42*E* − 01 (±1.19*E* − 01)	1.79*E* − 01 (±8.28*E* − 02)	1.86*E* − 01 (±9.83*E* − 02)	5.84*E* + 00*
AspectRatio	1.51*E* + 00 (±4.77*E* − 02)	1.60*E* + 00 (±4.40*E* − 02)	1.62*E* + 00 (±4.14*E* − 02)	1.23*E* + 01*
Roundness	6.88*E* − 01 (±1.73*E* − 02)	6.56*E* − 01 (±1.41*E* − 02)	6.50*E* − 01 (±1.29*E* − 02)	1.29*E* + 01*
Solidity	8.48*E* − 01 (±7.22*E* − 03)	8.41*E* − 01 (±7.98*E* − 03)	8.32*E* − 01 (±6.66*E* − 03)	8.87*E* + 00*
CytoplasmLength (*μ*m)	6.16*E* + 00 (±8.20*E* − 01)	1.29*E* + 01 (±1.16*E* + 00)	1.59*E* + 01 (±1.01*E* + 00)	1.72*E* + 02*
CytoplasmLength_SD_	2.10*E* + 00 (±4.06*E* − 01)	5.75*E* + 00 (±7.69*E* − 01)	8.47*E* + 00 (±7.17*E* − 01)	1.68*E* + 02*

*It indicates features whose null hypothesis was rejected with *F-*value > *F*_0.01_(2,237).

**Table 13:** LSDtest for *F-*test of nonepithelial nuclei features.

Nonepithelial nuclei features	LSD test			
	$\left|\bar{N}-\bar{G1}\right|$	$\left|\bar{N}-\bar{G2}\right|$	$\left|\bar{G1}-\bar{G2}\right|$	LSD-value

Area (*μ*m^2^)	4.08*E* + 00*	1.66*E* + 00	2.42*E* + 00*	2.23*E* + 00
Perimeter (*μ*m)	2.89**E** + 00*	1.81**E** + 00*	1.09**E** + 00*	9.34**E** − 01
Width (*μ*m)	9.75**E** − 01*	4.68**E** − 01*	5.07**E** − 01*	2.97**E** − 01
Height (*μ*m)	5.41*E* − 01*	3.92*E* − 01*	1.49*E* − 01	3.34*E* − 01
MajorAxis (*μ*m)	1.10**E** + 00*	7.04**E** − 01*	3.99**E** − 01*	2.49**E** − 01
MinorAxis (*μ*m)	—	—	—	—
Circularity	6.66*E* − 02*	5.68*E* − 02*	9.75*E* − 03	1.62*E* − 02
Feret's diameter (*μ*m)	1.22**E** + 00*	7.66**E** − 01*	4.57**E** − 01*	2.98**E** − 01
Skewness	—	—	—	—
AspectRatio	3.24*E* − 01*	2.60*E* − 01*	6.40*E* − 02	7.24*E* − 02
Roundness	7.64*E* − 02*	6.60*E* − 02*	1.04*E* − 02	1.67*E* − 02
Solidity	1.92*E* − 02*	1.88*E* − 02*	3.46*E* − 04	6.40*E* − 03

*It indicates that the absolute pairwise difference is greater than LSD value.

**Table 14:** LSD test for *F*-test of lumen features.

Lumen features	LSD test			
	$\left|\bar{N}-\bar{G1}\right|$	$\left|\bar{N}-\bar{G2}\right|$	$\left|\bar{G1}-\bar{G2}\right|$	LSD value
Area (*μ*m^2^)	2.99**E** + 04*	8.14**E** + 04*	5.15**E** + 04*	2.39**E** + 04
Perimeter (*μ*m)	6.01**E** + 02*	1.25**E** + 03*	6.46**E** + 02*	2.95**E** + 02
Width (*μ*m)	8.69**E** + 01*	1.80**E** + 02*	9.27**E** + 01*	4.93**E** + 01
Height (*μ*m)	6.05**E** + 01*	1.26**E** + 02*	6.56**E** + 01*	3.32**E** + 01
MajorAxis (*μ*m)	1.07**E** + 02*	2.08**E** + 02*	1.01**E** + 02*	5.34**E** + 01
MinorAxis (*μ*m)	5.91**E** + 01*	1.37**E** + 02*	7.80**E** + 01*	3.34**E** + 01
Circularity	3.90*E* − 03*	3.18*E* − 03*	7.25*E* − 04	3.13*E* − 03
Feret's diameter (*μ*m)	2.22**E** + 02*	4.54**E** + 02*	2.32**E** + 02*	1.09**E** + 02
Skewness	9.41**E** − 01*	1.20**E** + 00*	2.61**E** − 01*	2.48**E** − 01
AspectRatio	1.63*E* − 01*	1.34*E* − 01	2.90*E* − 02	1.34*E* − 01
Roundness	—	—	—	—
Solidity	—	—	—	—
AtypiaRatio	2.21**E** − 01*	4.17**E** − 01*	1.97**E** − 01*	7.99**E** − 02
#AtypiaRegions	2.40**E** + 00*	4.76**E** + 00*	2.36**E** + 00*	6.90**E** − 01
RMSAA	7.82**E** + 00*	1.77**E** + 01*	9.92**E** + 00*	3.77**E** + 00
TSAV (rad)	8.45**E** + 03*	2.59**E** + 04*	1.74**E** + 04*	5.88**E** + 03

*It indicates that the absolute pairwise difference is greater than LSD value.

**Table 15:** LSD*-*test for *F-*test of epithelial nuclei features.

Epithelial Nuclei Features	LSD-test			
	$\left|\bar{N}-\bar{G1}\right|$	$\left|\bar{N}-\bar{G2}\right|$	$\left|\bar{G1}-\bar{G2}\right|$	LSD-value
Area (*μ*m^2^)	1.04**E** + 01*	6.19**E** + 00*	4.26**E** + 00*	3.91**E** + 00
Perimeter (*μ*m)	3.92*E* + 00*	2.92*E* + 00*	1.00*E* + 00	1.41*E* + 00
Width (*μ*m)	1.07**E** + 00*	6.07**E** − 01*	4.66**E** − 01*	3.89**E** − 01
Height (*μ*m)	1.02*E* + 00*	7.60*E* − 01*	2.65*E* − 01	4.27*E* − 01
MajorAxis (*μ*m)	1.21**E** + 00*	7.96**E** − 01*	4.19**E** − 01*	3.71**E** − 01
MinorAxis (*μ*m)	5.30*E* − 01*	2.19*E* − 01	3.12*E* − 01*	2.95*E* − 01
Circularity	3.96*E* − 02*	5.35*E* − 02*	1.38*E* − 02	2.36*E* − 02
Feret's Diameter (*μ*m)	1.36*E* + 00*	9.36*E* − 01*	4.22*E* − 01	4.34*E* − 01
Skewness	1.64*E* − 01*	1.56*E* − 01*	7.53*E* − 03	1.40*E* − 01
AspectRatio	9.12*E* − 02*	1.11*E* − 01*	1.95*E* − 02	6.18*E* − 02
Roundness	3.19*E* − 02*	3.78*E* − 02*	5.90*E* − 03	2.07*E* − 02
Solidity	7.74*E* − 03	1.65*E* − 02*	8.74*E* − 03	1.02*E* − 02
CytoplasmLength (*μ*m)	6.78**E** + 00*	9.78**E** + 00*	3.00**E** + 00*	1.40**E** + 00
CytoplasmLength_SD_	3.65**E** + 00*	6.37**E** + 00*	2.72**E** + 00*	9.05**E** − 01

*: It indicates that the absolute pairwise difference is greater than LSD-value.

## Appendices

In this paper, we use PIP method [46, 47] to find critical points of atypia-amplitude signature. However, the existing PIP method may not be able to find points that variation of the lumen boundary becomes more complex because it detects a fixed number of critical points. Therefore, we introduce the modified PIP detection method that finds all critical points of given atypia-amplitude signature. The modified PIP detection method is processed by the following two steps: (1) finding all PIPs of atypia-amplitude signature and (2) postprocessing for eliminating unnecessary PIPs.

### A. Finding All PIPs of Atypia-Amplitude Signature

The PIP method finds critical points referred to as PIP (Perceptual Important Points) that represent important trends of time series data. In this paper, the existing PIP detection algorithm that detects a fixed number of PIPs has been modified to find all critical points in the atypia-amplitude signature. The modified PIP detection algorithm detects all PIPs with maximum vertical distances (VDs) [46, 47] above *Threshold* between adjacent PIPs. PIP_*Detection_For_Atypia_Amplitude_Signature* (Algorithm 2) shows the modified PIP detection algorithm in this paper.

As inputs, Algorithm 2 takes a sequence of points (denoted by *amp_list*) that forms the atypia-amplitude signature and the threshold (denoted by *T*) to detect PIPs. The algorithm output is a sequence of the detected PIPs (denoted by *pip_list*). The first step of Algorithm 2 initializes *pip_list* to the first point of *amp_list* (Algorithm 2, line 3). After that, all PIPs in a given *amp_list* are detected by *Sub_PIP_Detection* algorithm (Algorithm 3).

The *Sub_PIP_Detection* (Algorithm 3) finds a PIP within a given range of *amp_list*. The first and second inputs of the algorithm are start-index (denoted by *s*) and end-index (denoted by *e*) for the range of the *amp_list* to detect a PIP. Algorithm 3 first finds the location that has the maximum VD value in a given range (Algorithm 3, line 1). Then if the VD value of the found location is greater than a threshold *T*, the location is used as a pivot. The given range of the algorithm is split into two ranges, and the *Sub_PIP_Detection* (Algorithm 3) is called with the new two ranges (Algorithm 3 lines 5–7). If the VD value is less than *T*, the partition of the range is stopped and the point at end-index *e* of the given range for the algorithm is added to *pip_list* (Algorithm 3, line 9). The *Sub_PIP_Detection* (Algorithm 3) is recursively called until all PIPs satisfying the threshold condition are detected in *amp_list* (Algorithm 4).

The distance metric VD used in the PIP detection is the vertical distance between the test point and the line connecting the two adjacent PIPs. That is, VD at a PIP *p*_*i*_(*x*_*i*_, *y*_*i*_) between two adjacent PIPs, *p*_*s*_(*x*_*s*_, *y*_*s*_) and *p*_*e*_(*x*_*e*_, *y*_*e*_), is as follows:

(A.1) VD(ps,pi,pe)=|yc−yi|=|(ye−ysxe−xs×(xc−xs)+ys)−yi|,

where *y*_*c*_ is the value of a linear function determined by two points, *p*_*s*_ and *p*_*e*_, when *x*_*c*_ = *x*_*i*_ is given.  Figure 14 shows the VD between the line connecting the two adjacent PIPs (*p*_*s*_ and *p*_*e*_) and the test point *p*_*i*_.

### B. Postprocessing for Eliminating Unnecessary PIPs

Figure 15(a) shows the PIPs identified by the modified PIP algorithm (Algorithm 2). But some unnecessary PIPs can be found because the modified PIP algorithm identifies all PIPs satisfying the condition that the maximum VD is greater than a threshold. That is, several PIPs can be found where the change of the big trend has not occurred, as shown in Figure 15(a). These PIPs are points that have a trend in the same direction. To remove such unnecessary PIPs, postprocessing for the identified PIPs (*pip_list*) by the modified PIP algorithm (Algorithm 2) is performed. The postprocessing algorithm for PIPs is as Algorithm 5. The* Post_Processing_PIPs* (Algorithm 5) assesses whether each PIP of *pip_list* identified by Algorithm 2 is a maxima or minima when trend is reversed (Algorithm 5, lines 4–10). Then, if a PIP of the *pip_list* is not a maxima or minima, the PIP is removed in the *pip_list* (Algorithm 5, lines 11–14).  Figure 15(b) shows the result of Algorithm 5 for Figure 15(a).
